# “Catch 22”: Biosecurity awareness, interpretation and practice amongst poultry catchers

**DOI:** 10.1016/j.prevetmed.2017.04.002

**Published:** 2017-06-01

**Authors:** Caroline Millman, Rob Christley, Dan Rigby, Diana Dennis, Sarah J. O’Brien, Nicola Williams

**Affiliations:** a Department of Economics, University of Manchester, Manchester, UK; b Department of Epidemiology and Population Health, Institute of Infection and Global Health, Leahurst Campus, University of Liverpool, UK; c NIHR Health Protection Research Unit in Emerging and Zoonotic Infections, University of Liverpool, Liverpool, UK; d NIHR Health Protection Research Unit in Gastrointestinal Infections, University of Liverpool, Liverpool, UK

**Keywords:** Campylobacter, Biosecurity, Hazard awareness, Poultry, Catching crew

## Abstract

Campylobacter contamination of chicken on sale in the UK remains at high levels and has a substantial public health impact. This has prompted the application of many interventions in the supply chain, including enhanced biosecurity measures on-farm. Catching and thinning are acknowledged as threats to the maintenance of good biosecurity, yet the people employed to undertake this critical work (i.e. ‘catchers’) are a rarely studied group.

This study uses a mixed methods approach to investigate catchers’ (n = 53) understanding of the biosecurity threats posed by the catching and thinning, and the barriers to good biosecurity practice. It interrogated the role of training in both the awareness and practice of good biosecurity. Awareness of lapses in biosecurity was assessed using a Watch-&-Click hazard awareness survey (n = 53). Qualitative interviews (n = 49 catchers, 5 farm managers) explored the understanding, experience and practice of catching and biosecurity.

All of the catchers who took part in the Watch-&-Click study identified at least one of the biosecurity threats with 40% detecting all of the hazards. Those who had undergone training were significantly more likely to identify specific biosecurity threats and have a higher awareness score overall (48% compared to 9%, p = 0.03). Crucially, the individual and group interviews revealed the tensions between the high levels of biosecurity awareness evident from the survey and the reality of the routine practice of catching and thinning. Time pressures and a lack of equipment rather than a lack of knowledge appear a more fundamental cause of catcher-related biosecurity lapses. Our results reveal that catchers find themselves in a ‘catch-22′ situation in which mutually conflicting circumstances prevent simultaneous completion of their job and compliance with biosecurity standards.^3^ Hence, although education about, and enforcement of, biosecurity protocols has been recommended, our findings suggest that further reforms, including changing the context in which catching occurs by improving the equipment and other resources available to catchers and providing more time for biosecurity, will be essential for successful implementation of existing biosecurity protocols.

## Highlights

• Barriers to good biosecurity during catching are investigated using mixed methods.
• Awareness of biosecurity is assessed using a Watch-&-Click hazard awareness survey.
• Time pressure and lack of equipment are important causes of lapses in biosecurity.

## 1 Introduction

Recently published figures on the levels of *Campylobacter* contamination among chickens on sale in the UK have intensified the pressure on the poultry industry to tackle the ‘*Campylobacter* problem’. The joint UK government and industry target aimed to reduce the proportion of birds most heavily contaminated with *Campylobacter* to <10% by 2015 (FSA, 2013b), but the UK Food Standards Agency (FSA) analysis in 2014-15 (FSA, 2015c) indicated 73% of whole birds (chickens) were contaminated with *Campylobacter*, with 19% contaminated with >1000 cfu/g. A further, year-long survey has been commissioned by the FSA, showing some limited improvements in the most heavily contaminated (15% > 1000 cfu/g) in the first quarter of the survey (FSA, 2015b) and again in the second quarter (11% > 1000 cfu/g) (FSA, 2016).

Industry efforts to reduce levels of *Campylobacter* have targeted packaging technology (leak proof sealing and cook in the bag products), labelling (advising consumers not to wash chicken), factory processing (maximising the standards of hygiene using existing equipment or introducing new interventions such as steam treatment, surface chilling, and double scalding), live transport systems (improved crate sanitising), feed and farming systems (feed additions, heightened standards of biosecurity including the introduction of model farms and trials of no thinning) (FSA, 2015a). However, despite the range of initiatives and the many innovations identified and/or introduced *Campylobacter* contamination levels remain stubbornly high.

A major focus for control has been at the start of the supply chain, in the belief that interventions here offer the potential for prevention of, or reductions in the extent of, flock colonisation leading to a reduction in *Campylobacter* contamination through processing and retail. Although biosecurity requirements were included in the Red Tractor standard (a UK assurance scheme) in 2011, there has been little, if any, observed impact on *Campylobacter* contamination rates. The FSA suggested that “The lack of impact of this change might have been because the new requirements of the standard were flawed, or because they have not been applied by producers with sufficient consistency to be effective” (FSA, 2013a p. 4). As a result of this improved application of biosecurity-based interventions on the farm continues to be a focus of efforts to limit *Campylobacter* in the food chain.

Newell et al. (2011) summarised the literature relating to biosecurity-based interventions on farms, highlighting “a paucity of detailed understanding of both the sources of flock infection and those measures which might be effective for the prevention of flock positivity” (Newell et al., 2011pg 8614). They concluded that sample design compromised much of the literature with no clear transmission route identified to target interventions. Additionally, they determined that sustaining the rigorous application of biosecurity measures is fraught with difficulty and is only possible in conjunction with farm worker education and incentives. More recent work with so called ‘model farms’ (JWG, 2014) suggests on-farm biosecurity can be effective, as not all sheds became positive with *Campylobacter* on farms with good between-shed biosecurity, and that fewer sheds became positive on farms using the higher biosecurity standards.

One widely reported *Campylobacter* risk factor for broiler flocks is the process of thinning and subsequent house clearance (Allen et al., 2008, Newell et al., 2011, Ridley et al., 2011, Koolman et al., 2014, Smith et al., 2016). Whilst it is not yet clear if this relationship results from the effects of associated stress and bird age, or the breach in biosecurity that occurs because of thinning, the catching personnel, their biosecurity practices and the equipment that they use have come under increasing scrutiny and audit. For example, the Red Tractor standard was recently revised (October 2014) to include more requirements for catchers regarding biosecurity and the need for specific biosecurity training of catching personnel. Such training is conducted and recorded under the auspices of the Poultry Passport scheme (established 2008).

This paper investigates catchers’ understanding and experience of key biosecurity threats posed by (poor conduct of) the catching/thinning process, and whether differences in awareness differ over observable characteristics of the catchers. It investigates the barriers to good biosecurity practice in the catching process, and interrogates the role of training in both the awareness and (self-reported) practice of good biosecurity procedures on farm. These research questions are investigated through a mixed methods approach. The ability of catchers and catching team managers to identify biosecurity hazards was examined using a Watch-&-Click Hazard Awareness survey. Qualitative individual and group interviews were conducted with participants from these groups as well as a small number of farmers in order to assess their understanding, experience and practice of catching and of biosecurity.

## 2 Materials and methods

### 2.1 Watch-&-Click hazard awareness survey

#### 2.1.1 Questionnaire design

A Watch-&-Click survey was conducted with catchers and team leaders, comprising of a section of interactive video and additional questions. This real-time test was used in an earlier study relating to domestic food hazard awareness (Millman et al., 2015). Using this method, a film is embedded in a web-based interface to allow respondents to identify hazards, by clicking or tapping a tablet whenever a hazard was perceived to be evident on the screen – this response occurs in real-time as the film plays. These clicks are then turned into click response data (total number of hazards, correct and incorrect hazards identified) for interrogation alongside additional questions.

In this study, the film footage showed catchers carrying out thinning on a broiler farm with 7 deliberate mistakes, hazards or lapses in biosecurity (Table 1). Additional data (or characteristics) on demographics and experience was also collected from each respondent e.g. biosecurity training, employment status (self-employed/company employed) and (travelling) distance to the first farm of the day. Individual feedback was automatically generated for the respondents completing the survey, to show the hazards that they identified correctly and those missed, highlighting the importance of the biosecurity protocols.

**Table 1:** Biosecurity hazards and their identification by catchers (n = 53), including comparison of responses for those with and without (self-reported) biosecurity training. The number in each cell is the percent of respondents that correctly identified each hazard.

Hazards			Hazard identification (%)			
Description	Short name	Still from film	Overall n = 53	With Training n = 42	No training n = 11	PR Test^a^ p value
The catching crew wear clothes from another farm	clothes		70	76	45	0.048*
The catching forklift is not sanitised before going onto farm	Forklift		75	83	45	0.009**
Dirty clothing and boots are put on from the back of the catching van	Dirty clothes		72	71	73	0.932
Boots are not dipped on entry to the shed^b^	Dip		96	100	82	0.005**
The modules/transport crates are dirty	Crates		87	93	64	0.011*
The forklift is not sanitised before entering another shed	Between sheds		77	83	54	0.042*
The catching crew sit in their van for their break	Break		93	95	82	0.134

a PR Test = Test of proportions between those with and without (self-reported) biosecurity training* < 0.05; ** < 0.01.

b The boot dip was not situated at the entry to the shed as required for biosecurity – the location along a wall is also not permitting proper use. The hazard showed individuals not using this boot dip.

#### 2.1.2 Recruitment

Catchers and catching team managers were recruited from across the poultry catching industry in England to take part in the Watch-&-Click survey. Snowball sampling was used to reach different sectors of the industry, by sending emails to individuals across the poultry industry, via individual poultry companies, retailers, the National Farmers Union (NFU), British Poultry Council (BPC), Red Tractor and veterinary practices. However due to the low catcher response rate, five broiler farms were also visited (during catching) and catchers asked to complete the survey during break times. The broiler farms were located across three geographical areas (East Anglia, Lincolnshire, Midlands) and chosen in order to access catching crews and farmers from different companies. Catching crews travelled to farms from the North of England, Lincolnshire, East Anglia, Midlands and South West England.

#### 2.1.3 Data analysis

This survey approach was used to investigate i) how many biosecurity lapses individuals would identify – Hazard identification score, ii) which lapses would be more/less likely to be identified − identification of individual hazards and iii) variation in biosecurity awareness.

To investigate any variation in awareness, the impact of characteristics on the aggregate identification scores was assessed using a right censored Poisson model (Hilbe and Judson, 1999), with the distribution model capped at 7 hazards. Multiple correspondence analysis (MCA) and Hierarchical cluster analysis (HCA) (Husson et al., 2011) were applied in order to summarise the relationships between the seven hazards, in terms of their identification by study participants. Two additional supplementary qualitative variables (“company” and “no training”) and one quantitative variable (“total score”) were included. The effect of training on total score and the association between MCA dimensions and identification of individual hazards was assessed using the v-test, with a v-test > |2| indicative of a statistically significant effect. MCA and HCA were undertaken using R v3.2.2 (R Core Team, 2015) and the FactormineR library.

### 2.2 Group and individual interviews

#### 2.2.1 Interview selection

Nine group interviews were conducted with catching teams on five broiler farms to explore themes of biosecurity from the perspective of catching. Individual interviews were also conducted with five farm managers and four catching crew team leaders. The discussions took place during breaks or when crews were not under time pressures that may affect their responses.

#### 2.2.2 Interview design and analysis

A single interviewer undertook all interviews, and a topic guide was used to assist the interviewer in ensuring that key areas were covered. Topic areas included: training and level of experience; definitions and importance of biosecurity; training; catcher’s role in farm biosecurity; difficulties practicing biosecurity; ways biosecurity practices could be made easier; perceptions of biosecurity standards for individual and farmers; and, supervision of catching and biosecurity on farms. However, in keeping with qualitative research methods, the order in which these areas were discussed was determined by the participant(s) of each interview and additional relevant topics were pursued, with the aim that the participants could discuss these issues from their own perspectives and in their own words. All interviews and focus groups were audio-recorded, transcribed and anonymised. Thematic analysis (Braun and Clarke, 2006) was used to assess the transcripts to highlight minor and major themes. This commenced with line-by-line coding of transcripts. Subsequently, codes were reviewed in order to identify overarching themes.

Approval for the two elements of the study was provided by The University of Liverpool (VREC249) and The University of Manchester (14244) Research Ethics Committees. Signed consent was obtained from individuals participating prior to taking part in the interviews and focus groups, at which point the opportunity to opt out of being recorded was presented.

## 3 Results

### 3.1 Watch-&-Click survey

#### 3.1.1 Study population

Fifty-three catchers and catching team managers were recruited to take part in the Watch-&-Click survey. Ninety percent were British nationals, with a mean age of 38 years. Eleven percent of the sample had worked as a catcher for <1 year, 27% for 1–5 years and 62% for 5–10 years.

With regard to training, 79% (n = 42) reported that they had received some form of biosecurity training (either informal or formal in format), with 21% (n = 11) reported receiving none. Of the people who could remember when the training occurred, 87% said it was over 3 months ago and 73% said it specifically mentioned *Campylobacter*. Poultry passports have been in use within the industry since 2008 to provide a consistent level of training as well as a means of recording this for those working in the poultry sector. Although not mandatory, the training recorded within the passport is a requirement of the Red Tractor Scheme; 13% of individuals said that they held a poultry training passport, 38% were unsure what it was or if they had one and 49% stated that they did not hold one.

#### 3.1.2 Watch-&-Click responses

##### 3.1.2.1 Hazard identification scores

**Fig. 1:**
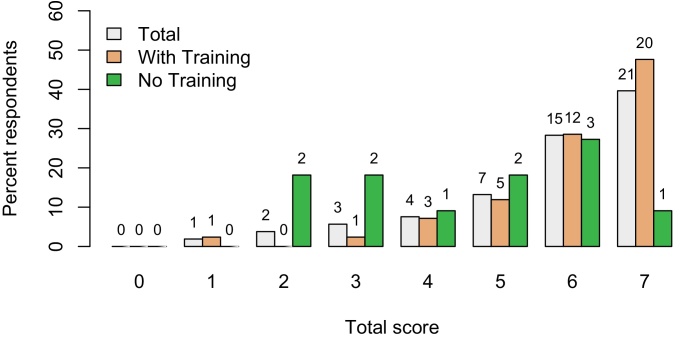
Distribution of total hazard identification scores among 53 catchers, and among those who stated that they had previously received biosecurity training (‘With training’, n = 42) and those reporting never having received training (‘No training’, n = 11). Total score represents the number of hazards identified by each respondent, out of a maximum of 7. The numbers above each bar indicate the number of individuals within that category.

Fig. 1 shows the distribution of the hazard identification scores overall, and for people who stated that they had had biosecurity training and those without training. All participants identified at least one of the hazards, with 40% of the respondents detecting all of the hazards. Significantly more of those who had received training, 48%, could identify all 7 hazards, compared to only 9% of those without training (Fisher’s exact test p = 0.03). The mean score for respondents with training, compared with no training, was 6.02 (sd = 1.32) and 4.45 (sd = 1.75), respectively.

##### 3.1.2.2 Identification of individual hazards

All hazards were identified by at least 70% of respondents (Table 1). The most commonly identified hazards were the catchers not using the foot dip (Dip, 96%), debris on the modules/crates (Crates, 87%) and the catchers taking their break in their crew van (Break, 93%). The least commonly spotted hazards were clothes being worn from another farm (Clothes, 70%), dirty clothes being put on from the back of the crew van (Dirty clothes, 72%), the forklift going straight onto the farm without cleaning (Forklift, 75%) and the forklift going between sheds without any cleaning or sanitisation (Between sheds, 77%).

##### 3.1.2.3 Explaining variation in hazard awareness

Further analysing the individual hazards (Table 1), the proportion of respondents that identified the ‘Forklift’ and ‘Dip’ hazards was significantly different at p < 0.01 (Forklift, p = 0.009; Dip, p = 0.005) between individuals that had been trained and those that had not, whilst ‘Clothes’, ‘Crates’ and ‘Between sheds’ were significantly different at p < 0.05 (Clothes, p = 0.048; Crates, p = 0.011; Between sheds, p = 0.042).

Estimation of the right censored Poisson count model permitted the investigation of the impact of respondent characteristics (biosecurity training, age, employment status, length of service) on individuals’ hazard identification scores.^4^ Using the Poisson model, only training was found to have a significant association, with an individual with no biosecurity training likely to identify, on average, 2.4 fewer hazards than a person who had received biosecurity training (p = 0.03).

**Fig. 2:**
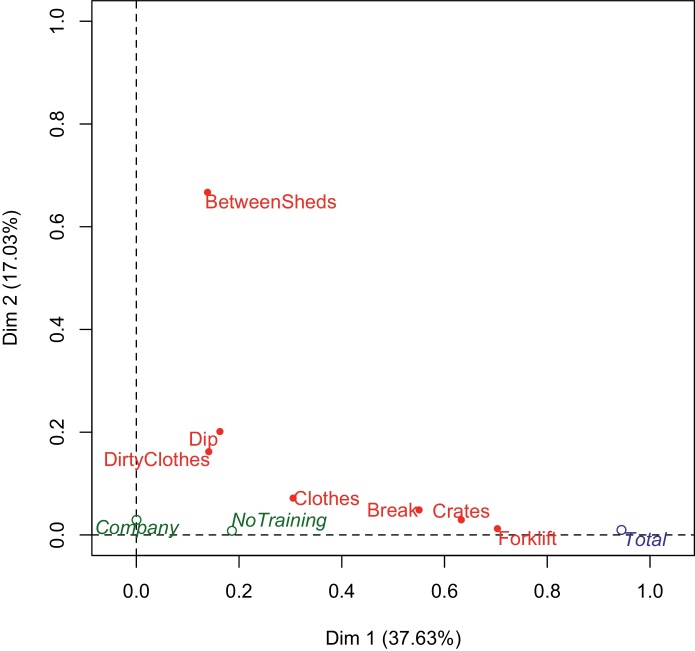
Representation of the 7 biosecurity hazards (red) on a plane defined by the two main dimensions identified using Multiple Correspondence Analysis. The axis values are the square of the correlation coefficients between the hazard and the dimension and hence are a measure of the quality of the projection of the hazard on the dimension. Hence, ‘Forklift’ is strongly related to dimension 1 and ‘Between sheds’ with dimension 2. Among the supplementary variables (blue indicates categorical variables and green continuous variables), total biosecurity score (‘total’) is strongly explained by dimensions 1 and training (‘No training’) less so, while ‘company’ is not influenced by either dimension. (For interpretation of the references to colour in this figure legend, the reader is referred to the web version of this article.)

Multiple Correspondence Analysis (MCA) was used to explore the co-identification of hazards and to relate this to respondent training. The first 3 dimensions identified using MCA accounted for approximately 71% of the variance in the data (36.6%, 17.0% and 16.6%, respectively). Dimension 1 was significantly associated (|v test| > 2) with all hazards (Table 2; Fig. 2), but was particularly influenced by individuals’ responses to ‘Forklift’, ‘Crates’ and ‘Break’. Dimension 1 accounted for almost all variation in the total hazard identification score (Fig. 2). Dimension 2 was significantly associated with identification of ‘Dip’, ‘Between sheds’ and ‘Dirty clothes’, while dimension 3 was significantly associated with ‘Dip’, ‘Dirty clothes’ and ‘Clothes’. Training was significantly associated with dimension 1 (Table 2, v test = −3.1), but not dimension 2 (v test = 0.008) or dimension 3 (v test = 0.05). Company was not associated with any dimension.

**Table 2:** Results of Multiple Correspondence Analysis (MCA) and Hierarchical Clusters Analysis (HCA) of biosecurity hazard identification by catchers (n = 53). The greater the contribution of a hazard to a dimension the greater its influence on this dimension. The significance of each hazard to each dimension is indicated by the v test, with |v test| > 2 indicative of a significant association (highlighted in bold). The percent of catchers correctly identifying each hazard is provided for each of the three main cluster groups suggested by Hierarchical Cluster Analysis.

			Dimensions identified using MCA						Percent correct identification of hazards within the main clusters identified using HCA		
Description	Short name	Identified hazard?	1		2		3		1	2	3
			Cont.^a^	v test	Cont.	v test	Cont.	v test	n = 35	n = 10	n = 8
The catching crew wear clothes from another farm	Clothes	False	**8.1**	**4.0**	4.2	−1.9	**5.4**	**−2.2**	83	50	38
		True	**3.5**		1.8		**2.3**				
The catching forklift is not sanitised before going onto farm	Forklift	False	**20.1**	**6.0**	0.8	0.8	0.0	−0.1	94	70	0
		True	**6.5**		0.3		0.0				
Dirty clothing and boots are put on from the back of the catching van	Dirty clothes	False	**3.8**	**2.7**	**9.7**	**2.9**	**36.3**	**−5.5**	74	100	25
		True	**1.5**		3.8		**14.3**				
Boots are not dipped on entry to the shed	Dip	False	**5.9**	**2.9**	**16.2**	**3.2**	**37.5**	**4.9**	100	100	75
		True	**0.2**		**0.6**		**1.5**				
The modules/transport crates are dirty	Crates	False	**20.8**	**5.7**	2.1	−1.2	0.9	0.8	97	80	50
		True	**3.2**		0.3		0.1				
The forklift is not sanitised before entering another shed	Between sheds	False	**4.1**	**2.7**	**43.3**	**−5.9**	0.6	0.7	100	0	75
		True	**1.2**		**12.7**		0.2				
The catching crew sit in their van for their break	Break	False	**19.3**	**5.4**	3.8	1.6	0.8	0.7	100	100	50
		True	**1.6**		0.3		0.1				

a Cont. = Contribution.

Hierarchical cluster analysis was used to identify clusters of respondents with similar hazard identification profiles; this approach suggested 3 main clusters (Table 2). Among individuals in cluster 1, 94% (33/35) had received training (78% of people that had received training were in cluster 1). Cluster 1 members also correctly identified all or most (>90%) of the following hazards: ‘Between sheds’, ‘Break’, ‘Forklift’, ‘Crates’ and ‘Dip’; and 83% identified ‘Clothes’. All members of cluster 2 correctly identified ‘Dirty clothes’ ‘Break’ and ‘Dip’, and 70% and 80% identified ‘Forklift’ and ‘Crates’, respectively, but none identified ‘Between sheds’ and this cluster included 83% of people who failed to identify ‘Between sheds’. Forty percent (4/10) of cluster 2 members received training. Two-thirds (5/8, 67%) of cluster 3 received no training, accounting for 45% of people who received no training. All members of this group failed to identify ‘Forklift’ (accounting for 61% of all people who did not identify ‘Forklift’). Half (50%) and a quarter (25%) of cluster 3 failed to identify ‘Break’ and ‘Dip’, respectively, accounting for all people who did not identify these hazards. Three-quarters did not identify ‘Dirty clothes’. Approximately two-thirds of members of clusters 1 and 3 (21/35 and 5/8) worked for an integrated poultry company, compared to only 30% (3/10) for cluster 2.

Total score varied significantly between the categories, with the mean score in cluster 1 (6.5) being significantly higher (v-test = 5.2) and the mean score in cluster 3 (3.1) being significantly lower (v-test = −5.1) than the overall mean (5.7).

### 3.2 Interviews

Nine group interviews were conducted on five broiler farms. Each group interview included between four and six catchers. Individual interviews were also conducted with five farm managers and four catching crew team leaders (total number of participants = 54). All of the participants were men, with ages ranging from 18 years to mid-sixties.

Within the interviews the members of the catching teams highlight a distinction between the informal on-the-job training (that is seen by many to be the mainstay of successfully becoming a catcher) and formal ‘company’ training. Our analysis also reveals the ways in which concepts of biosecurity (whether gained through formal or informal means) become enacted in the day-to-day reality of catching. Hence, even where biosecurity practices were widely known (as was evident for many practices in the Watch-&-Click study) our analysis highlights the practical, real-world dimensions that require catchers to modify protocols and procedures in order to meet the demands of their job and that provide rationales for such modifications.

Catching was almost universally reported to be very ‘*physical work’*, described as being “*one of the hardest jobs in Britain” or “the hardest physical job you*’*ll ever do”* and is a job that is “*not for old people*”. Working in teams, usually of around 4–6 people, catchers collect 5000 to 6000 birds per hour, placing them in crates or ‘modules’, and moving the modules on to trucks for transportation to the factory. On top of the hard physical work, catching is often conducted under very difficult conditions. In summer the temperature in the sheds was reported to reach 30 °C and was described as “unbelievable” or “ridiculous”, and that high concentrations of ammonia can make breathing difficult. These conditions increased the desire to get the job done quickly, and affected the suitability of some biosecurity procedures. For example, standard protective clothing was seen as unsuitable for working in hot conditions. Other issues with clothing were also noted. For example, trousers were sometimes worn outside the boots, in order to prevent litter from entering the boots, but this then caused problems at the boot dips. Similarly, boots with good tread were viewed as important for health and safety, but made boot disinfection difficult.

### 3.3 Training

Catchers distinguished between formal training, consisting of talks, videos, manuals and other reading materials, and informal, on-the-job training. Individual catcher’s experience of training varied greatly. To some extent this was put down to when people started in the industry; those who had been working as catchers for many years often reported only learning their skills and knowledge from their teammates and from their own experience, whereas people who had more recently joined catching teams were more likely to have reported receiving formal training.

Interviewer: Did they give you training on biosecurity?

Catcher 1: […] They give you the theory. They give you paperwork and things like that. […]

Interviewer: Did you see a video on that as well?

Catcher 1: Not on my time, no. […]

Catcher 2: I think, yes. I had a video and the paperwork.

Catcher 1: Yes. That's the thing. They just joined the company a year or two ago and they saw the video. Where people who have been here for 7 or 10 years, there are people have been here 15 years, they never had a video.

However, this distinction in training, between new-hands and experienced catchers, was often not always clear and variation appeared also to be associated with the support provided to the team, with some teams readily able to access training, whereas for others training was absent or very limited:

Catcher: the company runs courses all of the time. So we tend to do – I mean we’ve only had one recently with regards Campylobacter. Over the years we’ve done many with regards to biosecurity.

− − −

Catcher 1: I’ve been here nine months now, and I’ve never done nothing. I was told, “Go in that shed, catch them chickens, and that’s your job.” That’s it.

Catcher 2: Basically in this job you learn yourself.

The catchers often questioned the value of training, raising issues related both to the skills of the trainers and the value of formal training itself. Often, catchers believed that the trainers lacked the knowledge and experience that they had already gained through the time they had served as catchers:

Catcher: the guy at the front knows less about it than what you do. (Laughter) It gets a bit – and that’s not me being arrogant. That’s just a fact.

− − −

Catcher: As I say, and some of the courses are taken by ex-professionals, who are like people who have like worked on a farm, but a lot of them are just taught by teachers. There’s nothing wrong with that, but they don’t have the same knowledge that we do, or understanding sometimes of the practicalities

Furthermore, catching and biosecurity were viewed as practical activities that could not adequately be taught through classroom-based approaches that focussed on ‘theory’:

Catcher: It’s a manual job so the only way to learn is to do it. You can watch as many videos as you want but you’ll not do it.

This distinction between the need for practical training with the provision of classroom-based courses, perhaps combined with a perceived lack of experience of the teachers, caused some catchers to view the training as something of a box-ticking exercise, which satisfied the needs of the company, but which did not equip catchers with the skills needed to undertake biosecurity procedures within the practical realities of their work as catchers.

Catcher: They show you, you’re not trained. They just go, “Here, sign that, sign that, sign that.”

With or without formal classroom-based training, learning about catching and about biosecurity practices was almost universally seen as an on-going process mediated through practice and on-the-job learning – one that is both linked to individual experience and to intra-team relationships. This idea is well-expressed in the following quotation where catching is compared to learning to drive a car and in which learning theory is simply a step towards being able to undertake the essential practical training obtained through experience and repetition.

Catcher 1: The lad that has been there longer – obviously you just learn off them.

Interviewer: So usually that’s the way they do it – anybody new – they just go with the experienced one and there in the job.

Catcher 1: Yes. It’s the best way. Rather than watching a video.

Catcher 2: Yes. You’re better off learning off your own experience.

Catcher 1: It’s like doing your driving isn’t it? Once you’ve passed your test then you learn to drive.

For team leaders, biosecurity training must be tailored to the needs of individuals, with considerable effort required to ‘force’ some members of the catching team to follow procedures, whereas for others it has become part of their working practice.

Team Leader: some of the guys are brilliant. They’re absolutely spot-on. They’re on it; they know exactly what they’re doing, and they know what they need to do. Others, it’s just like you’re pushing uphill all the time. It’s just an uphill struggle, but they do it in the end […] they do it under – not under pressure, but it’s really banging it home all the time. We’ll spend time with them; that’s what we’ll do. We’ll be out here on the job all the time, if it needs that, but generally, they are quite good, to be fair. They are quite good

This diversity in biosecurity practice within the catching team is at odds with the perception of many catchers, highlighted above, that biosecurity training is best obtained through observation and learning within the team. This team-based approach would suggest that the practices of team-members converge, over time, toward a standardised practice. However, the statement from the team supervisor highlights great diversity among team members and the work required to make some people follow protocols. In reality, learning from, and providing training to, members within a team is likely to be a feature of the within-team relationships that were frequently reported to be a key feature of a successful catching team.

Catcher: You’ve got to be a team. It’s no good having 5 men, 10 men, 12 men – if you’re not a team, it doesn’t work properly.

− − −

Catcher: 1: Yes some teams – like us lot – we all get on well together like I say, you know, you’ve got to work together, and if you don’t work together you might as well forget about it. […] You’ve got to get on well with everybody. […]

These relationships may, therefore, facilitate within-team training through informal discussion and observation, but are unlikely to be able to enforce adherence to a specific set of practices or protocol.

### 3.4 Protocol and practice

While catchers may claim to understand the concept and practices of biosecurity, and the related rules and procedures that are set out by the company and/or individual farms they are working on, they were also aware of the shortcomings of such procedures and the impacts of these on disease control.

These shortcomings arise through the process of biosecurity practice, equipment, the physical nature of catching and the environment in which it is performed, and issues of time (and other factors, undetected in our analyses, may also be at play). These concerns interact with each other, and coalesce with notions of what it means to be a ‘good catcher’, forcing reinterpretation and reformulation of standardised protocols for biosecurity taught through formal training, replacing these with locally derived (and necessary) on-the-job training that is responsive to the physical and organisational realities of the job.

Indeed, the *process* of thinning itself, as well as the number of times that it could take place within one flock, was viewed by many as contradictory to the goal of biosecurity procedures. The physical hygiene barrier system, in the form of the chicken shed, was broken as soon as the shed doors were opened and in some cases this breach could be repeated a number of times.

Catcher: We did it [thinning] last night and that’s the third stint in that shed […] They have been taken off the feed three times, for three thinnings.

Furthermore, the practice of biosecurity was inconsistently applied to the act of entering a shed; whilst catchers were asked to dip their boots in disinfectant solution on entry to the shed, the wheels of the forklift truck typically were not cleaned between when moving in and out of the shed (taking modules off and onto the live transporter).

A key issue, frequently raised by catchers, was the impact of *biosecurity equipment* (or, its absence) on their ability to enact biosecurity procedures. The experience of our participants varied greatly, with some reporting that only around half of farms had adequate cleaning equipment, and others suggesting that facilities were generally poorer on privately-owned farm, compared to company-owned farms. Inadequate facilities for washing, was a recurrent theme discussed by catchers. In some cases water for washing was not provided on a farm, or was only available through a single, perhaps inappropriately located hose with insufficient water pressure to clean boots, the forklift truck or other equipment.

Catcher: The hosepipe outside, that's all they've got for washing the forklift with before they go somewhere else or take it back to the yard. […] That's the only way they have of washing the forklift

Furthermore, this lack of adequate cleaning equipment could be compounded by a lack of other facilities, such as lighting.

Catcher 1: We finish around midnight every day, which is pitch black and some places don't even have illumination or very poor illumination.

Catcher 2: Outside of the sheds.

Interviewer: So it's difficult for you to clean?

Catcher 1: It's difficult to clean. You don't see anything.

Many catchers see provision of inadequate biosecurity equipment and facilities as an area of dispute with farmers and/or the company, as this equipment is necessary for them to perform their role as catchers;

Catcher: It should be there anyway. We shouldn’t be having to ask for it. It should be already done.

Farmers also reported frustration because they “….tried lots of things but nothing makes a difference….” leading to suggestions that there was little to be gained through rigid implementation of biosecurity procedures (as one farmer put it: “what’s the point?!”). Indeed, the changes to biosecurity practices over the years were sometimes seen as having no real impact in practice.

Farmer: My sheds aren’t any better than last year when I wore the same boots, or 10 years ago where you didn’t even have wellies….There’s no worse disease now than there was then. I don’t see any difference at all. I mean when I started it was leather boots, then we went onto wellies, then you went to shed-specific wellies. Then you went to a barrier system. I’ve double dipped. I’ve triple dipped. I’ve worn over boots and it’s never made a second’s worth of difference…

*Time* was a recurrent theme raised by catchers during discussions. The working day for catchers is often very long and their work is undertaken under near constant pressure to meet deadlines imposed by the factory. Typically, catchers will be picked up at home to travel, often for several hours, to a farm to commence catching. Among the catchers completing the Watch & Click survey, 84% reported that they travelled more than an hour to the first farm of that day, with 41% reporting over 90 min; only 16% reported that they travelled an hour or less. Once finished on that farm, the team may move on to one or two more farms in a single shift.

Catcher: Sometimes you can do 15 h in a day. You come back and you have ten hours off. You literally get home, shower, eat, sleep, wake up and get back to work, no social life.

The intersection between time pressures and the implementation of biosecurity practice was clearly evident in discussions. Catchers have a very specific period of time in which they must conduct the catch, and biosecurity procedures must be built into this time. Early completion of the task enables some extra downtime for the team, providing added strong incentive to work quickly. The time provided may not vary with the physical reality of the catch, such that the need for additional biosecurity requirements may not be built into the time available.

Catcher: Look at [name] now. Has he really got time to go and wash his forklift off? […] To put four mods in, to wash it off, to go and get the mods out– because they don’t get any extra time. If you’re on two sheds you get the same amount of time so they’ve got to get those 10 stacks done. I mean to do it properly, he’d have to disinfect that now to put his stacks in, to disinfect again to go and get the full ones out of shed one, to disinfect again to put the ones into shed two. It’s not going to happen. 10 min of time? You’re not going to put 40 min and unload the chicken. They don’t get an extra second to do it…

The catchers are also aware of the contradictions in biosecurity protocols and the reality of planning, citing examples of breaches in biosecurity in the order in which they visit farms (i.e. visiting a farm to conduct thinning after visiting one undergoing a full depopulation) and the complexities of multiple activities taking place on a farm at any one time.

Catcher: You’ve got people cleaning, us catching, you’ve got people on the site to wash […] What’s that all about?

The effects of time on the practice of biosecurity may be further influenced by inadequate equipment. The time available for cleaning was seen to be predicated on having suitable equipment to hand: the lack of such equipment required either extra work time, or only partial fulfilment of a biosecurity procedure.

Catcher: They give you 15 min between each shed.

Interviewer: Is that enough? I heard it takes about 40 min at least to clean up the forklift.

Catcher: To do it properly, yes but if you have really decent power, then you can do it at a faster pace.

− − −

Catcher 1: … sometimes you can be there 40–45 min washing the forklift because it’s crap.

Catcher 2: Sometimes the hose hasn’t even got a nozzle at the end, you’ve got to put your thumb over it to get any power.

− − −

The long workday also effects catchers’ ability and/or willingness to engage with training material.

Interviewer: Okay. What about … reading … materials, or a handbook or a manual – those sorts?

Catcher: You’ve also got to think – we travel to work … It takes us two hours to get down here, and two hours to get back home, so that’s four hours … So it’s a long day.

Both the catchers and farm managers were quick to express the view that others in the chain were responsible for lapses in biosecurity. Farmers indicated that catching teams were at fault for breaking biosecurity:

Manager: they know on some farms they can get away with something and on other farms they can’t.

Catchers felt that the farm managers did not do enough to manage litter quality or make sure that they had the resources that they needed to carry out their job, whilst it was them, the catchers that took the blame for biosecurity issues.

Catcher: Not everybody follows the biosecurity procedures. We have to because people are watching us all the time because they’re always trying to blame this part of the industry…

Examples of breaches in biosecurity were readily offered by both farmers and catchers – these included the movement of bales into sheds, lack of overalls/changes for different farms, access for fitters and the lack of desire to want to change wellingtons at the hygiene barrier in the wet and winter. One participant stated that in their opinion farmers “stick to the rules 90% of the time”.

## 4 Discussion

The mixed methods approach used in this study provided an opportunity for the catchers and farmers to give some insight into their experiences, perceptions and understanding of biosecurity. At the same time, the use of the Watch-&-Click survey enabled data to be gathered from individuals on their awareness of specific biosecurity protocols.

Almost four-fifths of the catchers that took the Watch-&-Click test had received training. However, as this study did not use a random sample of catchers we are unable to estimate the extent to which this reflects the level of training among catchers in England and Wales. In 2006, it was estimated that 300–400 catchers worked in the broiler sector (Gittins and Canning, 2006). This same report, prepared for the Department for Environment, Food and Rural Affairs (DEFRA) and the UK FSA, concluded that biosecurity training was required for the catching industry. It is not clear if the high level of training seen, as part of the study, was in response to these recommendations. Additionally, the study was completed in the implementation period for a new biosecurity training clause in the Red Tractor standard (Red Tractor Assurance for Farms – Poultry Scheme, 2014). The new clause requires the catching team to be trained at induction and annually to minimise bird stress at depopulation and understand biosecurity issues, with the biosecurity standard operating procedure to include reference to *Campylobacter* controls. Despite reported levels of biosecurity training being high, it is worth highlighting that many participants in the current study lacked awareness of the Poultry Passport scheme, which provide a transferable training record for people involved in the poultry industry (www.poultrypassport.org). This suggests that, for many people, the training that has been undertaken has not been appropriately recorded and/or that the participants did not understand the role of the Passport.

The results from the Watch-&-Click study indicate that many of the catchers in the study had a high awareness of the biosecurity measures needed on farms and the ability to identify hazards when they are shown to them in real-time, with many participants successfully identifying all 7 hazards. There was evidence that biosecurity training improved hazard identification, with a significant effect of training on the ability to successfully identify all hazards (with half of trained participants recognising all hazards, compared to less than 10% of untrained participants). Additionally, a training effect was evident for the recognition of two specific hazards (Forklift not sanitised before going onto farm and Boots not dipped on entry to the shed). This correlates with the findings from Millman et al. (2015) which show that individuals with food safety training or knowledge are more likely to identify the hazards shown in a clip showing domestic food safety hazards. The MCA revealed that the majority of variation in total score was attributable to one dimension that was most influenced by identification of three hazards ‘Forklift’, ‘Crates’ and ‘Break’. Furthermore, this dimension was affected by training, which may suggest that information about these three biosecurity issues is covered more frequently in training. The impact of training on hazard identification was also illustrated using HCA, with cluster 1 which had the highest average score, including most of the people who had received training, compared to the lowest average score for cluster 3, which had the lowest proportion who had received training. The main cluster groups identified using HCA suggests sub-populations of catchers that vary in their ability to identify biosecurity hazards and in their experience of training.

The hazards that were used in the film footage were chosen to represent those listed by assurance schemes, commonly included in company biosecurity protocols and that may present a varied response in terms of ease of identification. Unfortunately due to the length of the film and difficulty in demonstrating (on film) some risk factors associated with catching (Newell et al., 2011), some risk factors such as the absence of hand washing were omitted. During the collection of data it was noted that procedures varied greatly on the use of gloves and wrist/arm guards (to prevent scratching) and the point at which hands were washed as set out in the Red Tractor Standard (Red Tractor Assurance for Farms – Poultry Scheme, 2014). Campylobacter has been identified on the hands of catching personnel(Newell et al., 2011), so this is an area of compliance that would warrant investigation. One of the hazards used, ‘Between sheds’, is not usually included in protocols but has been the subject of trials at some model farms. It is therefore of interest that this hazard was identified by a relatively high number of participants (77%). This may well be due to the focus of on-farm biosecurity, the awareness of some biosecurity contradictions (highlighted by individuals in the interviews), or simply the action of the film following the forklift providing a ‘clue’.

The Watch-&-Click tool was developed to provide video footage of an environment that is familiar to the catchers, reducing the observer bias that is often evident when individuals are watched within observational studies (Gill and Johnson, 1997). The use of video also creates a time pressure for the individual to respond using fast, automatic responses instead of deliberating the answer. In turn, it is hoped that the fast responses provided are that of knowledge and routine. Whilst there is no comparison with a more traditional survey, the results indicate that levels of knowledge are high, which was subsequently substantiated in the interviews.

Surveys have been carried out amongst the poultry farming community (although mostly in relation to avian flu) to assess the standards of biosecurity in place (East, 2007, Dorea et al., 2010, Racicot et al., 2011, Van Steenwinkel et al., 2011, Racicot et al., 2012, Ssematimba et al., 2013). However, few of these studies have used observation to evaluate the performance of the biosecurity practices, and therefore have not assessed compliance to biosecurity protocols. In contrast, Racicot et al. (2011) used video cameras to record biosecurity protocols in practice. These authors suggest that the effectiveness of the protocols depends on the consistency of their application by the farmers and farm workers, citing training as the key to improved standards.

However, further work by Racicot et al. (2012), also using video observation of poultry farm workers, found that level of education, duration of experience and personality traits, influenced compliance with biosecurity protocols. However, the association between experience and compliance was complex, with compliance greatest in those with 5–11 years experience and lowest in those with less than 5 years, or more than 24 years experience. They argued, following Park and Jung (2003), that the lower compliance among less experienced individuals may be due to a lack of, or ineffective, training, as people with limited experience rely to a greater extent on training rather than their own experience. In contrast, compliance among more experienced individuals may be associated with greater recognition of the consequences of non-compliance (Park and Jung, 2003). Racicot et al. (2012) also suggested that the increased compliance among workers with 5–11 years experience may be due, in particular, to the highly pathogenic avian influenza outbreak in 2004 in British Columbia, which led to a range of biosecurity initiatives. Hence, the relationship between training, experience and compliance is likely to be complex and vary with situation. As the study was not able to determine the detail of training conducted with the catchers, only the reported completion, it is unclear to what extent individuals received training on the principles and need for biosecurity versus the procedures. Nevertheless, these findings provide a point of departure for consideration of the distinction between formal and on-the-job training noted by participants in the current study and suggest this issue requires further investigation.

In the current study, whilst some individuals questioned the value of training, overwhelmingly the catchers knew many of the recommended biosecurity procedures, as evidenced by both the high scores on the Watch-&-Click survey and in the discussions during the interviews, suggesting that biosecurity training was more effective than realised by the catchers. However, the current study found that biosecurity protocols are often not followed even where knowledge is present. Hence, the key new insight from our study is that deviation from recognised procedures and protocols (i.e. lack of compliance), rather than being due to ignorance, is often a conscious decision to modify biosecurity practices in order to meet the demands of the job, within the practical and organisational constraints in which they operate, and were therefore usually seen as unavoidable compromises. Furthermore, the catchers’ and farmers’ experience of the messy and complex nature of catching made them question the value of rigid performance of biosecurity protocols, which contribute to a lack of motivation to comply with these. These findings build on those of others who report that, for example, cattle and sheep farmers lack of faith in the efficacy of farm-level biosecurity and concern that improved biosecurity will reduce autonomy (Gunn et al., 2008), that sheep and pig farmers generally feel that are doing all they can to minimise disease risk and that those practices not implemented are either not relevant or ineffective (Garforth et al., 2013). Catchers were well aware of contradictions between biosecurity procedures and the practicalities that arose due, for example, to the planning and scheduling of delivery of birds to the factories alongside additional agriculture operations to achieve that, including cleaning and farm management. For example, repeated thinning of sheds (as opposed to the recommended single thinning) and scheduling of thinning after depopulation of another farm (again, contrary to recommendations) were recognised by catchers and farmers as poor practise and breaches of companies’ own biosecurity protocols. These contradictions led catchers and farmers to openly question the protocols and the need for their own compliance with these.

Thus, rather than nonconformity with standardised biosecurity protocols being seen indicative of poor work practice amenable to education or enforcement, it was viewed by the catchers themselves as evidence of their resourcefulness and strong work ethic in the face of adverse conditions. These findings are in keeping with what Timmermans and Berg (1997) call ‘local universality’, which is the idea that standardised practices (such as are prescribed in protocols) can only be universal (i.e. work in different places and at different times) if they are locally adapted. We suggest that, whilst reduction or removal of the constraints to fulfilling the protocol (such as provision of extra time and equipment) may improve compliance, emphasis only on training and/or coercion is unlikely to be fully successful.

## 5 Conclusion

This study reveals that a lack of knowledge is unlikely to be the sole, or even the main, factor behind an apparent failure to of catchers comply with biosecurity protocols in poultry production systems in the UK. Many of the catchers included in this study were able to identify most or all of the hazards included in the Watch-&-Click survey. Furthermore, quite detailed knowledge of biosecurity protocols was often revealed during interviews. However, an important conclusion from this study is that (what might be considered to be) failure to comply with recognised procedures and protocols was usually reported by the catchers themselves as the result of deliberate or unavoidable action taken in order to meet the demands of the job.

In our view, catchers find themselves in something of a catch-22 (Heller, 1961) in which mutually conflicting circumstances prevent simultaneous completion of their job and compliance with biosecurity standards. Hence, although education about, and enforcement of, biosecurity protocols has been recommended (Gittins and Canning, 2006, Allen et al., 2008), our findings suggest that further reforms, including changing the context in which catching occurs by improving the equipment and other resources available to catchers and providing more time for biosecurity, may be essential for successful implementation of existing protocols.

## Disclaimer

The views expressed are those of the authors and not necessarily those of the NHS, the NIHR, the Department of Health or Public Health England.

## Footnotes

4 Results available from authors on request.
